# Effects of Al_3_Ni compound on localized plastic deformation and creep strain of Al/Si hypoeutectic material

**DOI:** 10.1038/s41598-023-50868-w

**Published:** 2024-01-18

**Authors:** Shakib Alsowidy, Belqueis Al-Asry

**Affiliations:** Department of Physics, Faculty of Science, Sana’a University, Sana’a, Yemen

**Keywords:** Materials science, Physics

## Abstract

The mechanical resistance of AS10/xNi hypoeutectic alloy with (x = 0.05% and 0.1%) has been investigated. Vickers hardness (HV) was determined for the samples before and after sintering. All samples were subjected to a compressive creep test at a constant temperature of 298 K and a constant load of 45 MPa. Creep parameters, such as creep rate, sensitivity (m), time exponent (n), β, and ɣ have been calculated and related to the Ni content. Microstructure investigation was conducted using the scanning electron microscope technique (SEMT). After sintering, the results showed that there was a significant improvement in the hardness with the addition of nickel. There is an increase in creep resistance as a result of the distribution of Al_3_Ni chemical compound across grain boundaries, which stops additional dislocation movement and hence reduces the creep rate.

## Introduction

**Figure 1:**
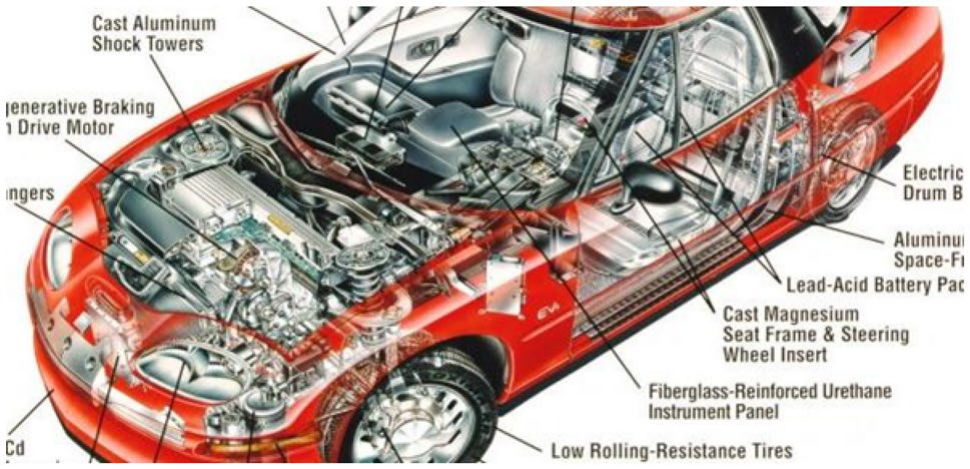
The car body is made of lightweight aluminum alloy^2^.

Previously, in order to withstand severe operating conditions, the structural materials of the automotive industry are made of cast iron and its alloys. Currently, the components of the structures of the automotive industry are mostly made of Al and its alloys^1^ (Fig. 1).

Modern structural industries require strong and lightweight metals due to a growing desire to minimize fuel consumption. These requirements are very important to the economy and environment-friendly^3^. So, these conditions must be taken into account. Aluminum and its alloys are currently the most popular light metals. The greatest Al advantages include its low density about (2.7 g/cm^3^) and high plastic deformation up to 30 percent^4^.

Aluminum alloys, such as Al/Si, Al/Mg, Al/Cu, etc., have various characteristics such as resistance to corrosion, mechanical strength, good castability, working at RT up to 400 °C, responsive to heat- treatment, and low cost^5^. All alloys mentioned above have α-Al dendrite phase. The spacing between dendrites is one of the important factors in determining the alloy properties like the strength of the alloy. As the spacing between main dendrites and secondary dendrites becomes smaller, the structure becomes fine^6^. The addition of an alloying element to the Al major produces secondary phases. These phases are intermetallic compound phases that reduce the secondary dendrite arms spacing (SDAS)^7^. On the other hand, heat treatment of metal is considered a favorable technique for enhancing characteristics of aluminum after casting and cold working^8^.

Aluminum Silicon alloys are commonly subjected to a variety of mechanical loads and relatively harsh environmental conditions when employed in structural applications^9^. These stresses can cause long-term irreversible plastic deformation before failure even if they are below the yield strength of aluminum alloys^10^. As a structural material has a high resistance to ambient environmental stresses as the service life has improved. Microstructure defects include dislocations and grain boundaries. Small grain size structure means a larger area of grain boundaries that act as barriers to the dislocation motion. So, the resistance to dislocation motion increases with increasing grain-boundary area^11^. It is reported that the strength of the material is related to the grain size by the following Hall–Petch equation^12^,

1 $$\sigma \text{=} {\text{k D}}^{{ - {1}/{2}}}$$

where σ is the yield strength is a constants and D is the average grain size. This means that decreasing grain size and increasing grain boundary area reflects on the strength of mater^13^. From those mentioned above, localized plastic deformation and mechanical plastic strain decrease as microstructure defects increase^14^.

However, in addition to the possibility of high dislocation concentration in pure aluminum that may produce after cold-working, pure Al has significantly poor mechanical properties, such as low hardness (~ 20 HB) and low yield strength (~ 70 MPa)^14^. The poor mechanical properties of this material are a result of microstructure defects^15^. To address all of these issues, the density of defects across the dendritic microstructure must be reduced dramatically. The goal of the current study is to improve the microstructure characteristics of the AS10 alloy in order to increase its mechanical strength and reliability factor by adding traces of nickel alloying element.

## Materials and methods

**Figure 2:**
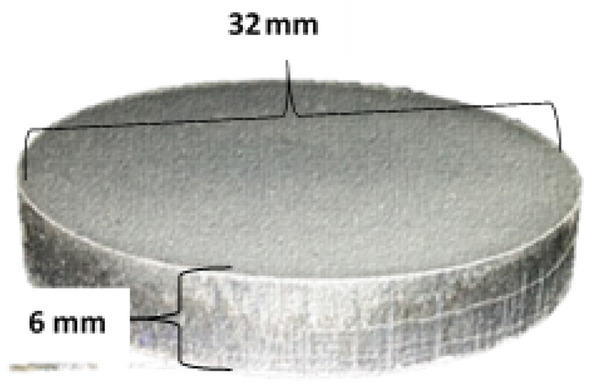
Compressed sample.

*Powder Aluminum (Al; 99.99%), Silicon (Si; 99.99%) and Nickel (Ni; 99.99%) have been chosen in this work. All materials were obtained from the BDH company (England) by Materials Science Laboratory at Sana’a University.* The nanopowder aluminium, silicon, and nickel were accurately weighted in balance to make the required composition as shown in the Table 1. The balanced elements were mixed in a powder form at room temperature. The balanced powder mixture was subjected to compression using a hydraulic pressure system with a constant load of 250 KN for 5 min for all three samples. The compacted circular disks obtained with dimensions of 6 × 32 mm is shown in Fig. 2. After that, the compressed samples were sintered in a controlled atmosphere furnace at about 773 K to allow the bounding of particles for each other.

**Table 1:** Chemical composition of the Al–Si–Ni alloys.

Alloy	Sample	Si (%)	Ni	Al
AS10	A_0_	10	0	Balance
AS10-0.05Ni	A_1_	10	0.05%	Balance
AS10-0.1Ni	A_2_	10	0.1%	Balance

## Characterization

**Figure 3:**
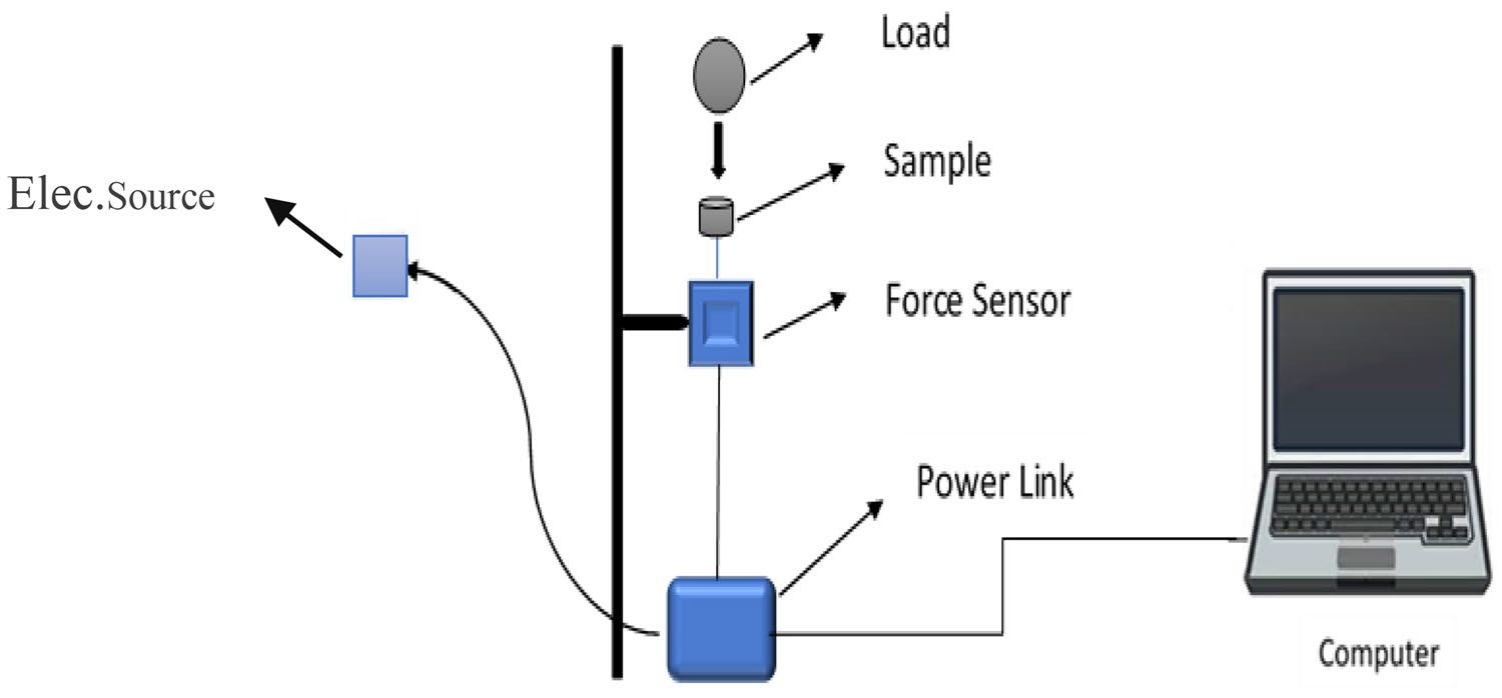
Schematic diagram of Vickers hardness tester.

The Vickers hardness test (localized plastic deformation resistance or impact force resistance) was performed using mechanical equipment designed in the Materials Science Laboratory at the Physics Department in the Faculty of Science, Sana’a University, Yemen, as shown in Fig. 3.

A compressive creep test was performed on all samples at a constant load of 45 Mpa and a constant temperature of 298 K by using a compression device (CMT4000,China).

Microstructure investigation was conducted using the scanning electron microscope technique (SEMT) (JEOL JSM 6510 Lv). Imaging has been done by irradiating samples with a highly accelerated electron beam. Secondary electrons (SE) and backscattered electrons (BSE) produced from the top and beneath the samples were detected and processed by scanning backscattered electron detector (SBED) and finally displayed as an image on the screen.

### Vickers hardness

In Vickers hardness testing, Pyramidal indenters methods are used and produce geometrically identical indentations^16^. On each specimen, at least three indentations were made and the average of these indentations served as the representative hardness value. The Vickers hardness number, HV is calculated using the following expression^17^:

2 $${\text{HV}} = {1}.{85}\frac{L}{{d^{2} }}$$

where L is the indentation load in kg and d is the mean diagonal of the indentation in mm.

### Transient creep strain

With regard to the transient interval of the creep curve, the transient creep strain is commonly touted by the familiar Friedel’s equation^18^:

3 $$\upvarepsilon_{{{\text{tr}}}} = \, \upbeta {\text{t}}^{{\text{n}}}$$

where Ɛ_tr_ and t are the transient creep strain and time. β and n are the transient creep parameters. The exponent n is determined from the slope of the relation between lnε_tr_ and lnt, while the coefficient β is calculated from the intercepts at lnt = 0 as clear from the Eq. (4):

4 $${\text{ln }}\upvarepsilon_{{{\text{tr}}}} = {\text{ ln}}\upbeta + \, n{\text{ ln}}t$$

### Steady-state creep stage

The applied stress provides a driving force for the dislocation movement and diffusion of atoms. As the stress is increased, the rate of deformation also increases. In general, it is found that

$$\dot{\upvarepsilon }_{{{\text{st}}}} \upalpha \upsigma^{n}$$

where n is termed the stress exponent.

By utilizing the relationship between the coefficient β and the strain rate of steady state creep (ε̇_st_), we are able to determine the exponent ɣ, as indicated in the equation^19^:

5 $$\upbeta = \upbeta_{0} (\dot{\upvarepsilon }_{{{\text{st}}}} )^{\upgamma }$$

By taking the logarithm of both sides of the equation:

6 $${\text{ln}}\upbeta = {\text{ ln}}\upbeta_{0} + \upgamma {\text{ln}}\dot{\upvarepsilon }_{{{\text{st}}}}$$

The equation governing the rate of steady state creep is:

7 $$\dot{\upvarepsilon }_{{{\text{st}}}} = {\text{A}}\upsigma^{n} {\text{exp}}\left( { - \frac{Q}{RT}} \right)$$

where, Q is the activation energy; n is the stress exponent; and *A* is a constant.

Strain rate sensitivity parameter (m) was calculated by the equation^20^:

8 $$m \, = \, \left( {\partial \ln \upsigma /\left( {\partial \dot{\upvarepsilon }_{{{\text{st}}}} } \right)} \right)$$

Parameter (**m**) is very important in characterizing structure superplastic deformation.

The activation energy is the energy needed to change the position, and its value is determined by the equation^21^:

9 $$Q = - R\left( {{\text{Ln}}\left( {\dot{\upvarepsilon }_{{{\text{st}}}} } \right)/{\text{Ln}}\left( {1/T} \right)} \right)$$

where T and R are temperature (Kelvin), and the gas constant, respectively.

## Result and discussion

### Mechanical properties

#### Vickers hardness measurements

Table 2 lists the Vickers hardness values of AS10-xNi specimens before and after sintering. The hardness increases from 67 to 86.9 at 0.05% Ni and also from 80 to 92.9 at 0.1% Ni. From Table 2, the highest hardness value at 0.1% Ni before sintering is 80 HV, while the highest hardness value at 0.1% Ni after sintering is 92.95 HV.

**Table 2:** Vickers hardness number (VHN) for alloys.

Material	Vickers hardness (Hv)	
	Before sintering	After sintering
A_0_	56.01	59
A_1_	67.37	86.98
A_2_	80.04	92.95

**Figure 4:**
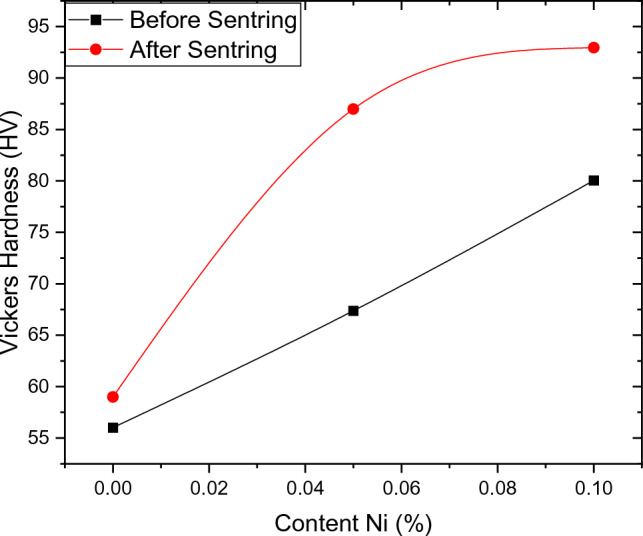
VH as a function of Ni-content.

From the Vickers hardness results, it is found that by increasing Ni-content, the HV significantly increases before and after sintering as shown in Fig. 4. From Al/Ni-binary phase diagram, it is clear that the maximum solubility of Ni into Al is 0.04% and most Ni constituent atoms react with Al atoms to produce Al_3_Ni chemical compound. By increasing Ni-content the amount of Al_3_Ni also increases as observed in images of SEM. The formation of a hard Al_3_Ni-IMC improves mechanical strength that is a very important characteristic in automotive Al-alloys^22,23^.

In the literature studies^24^ and like ceramic materials, Al_3_Ni chemical compound has a complex crystal structure (orthorhombic), and works as a dislocation slip resistance. So, the dislocation slip is destroyed and the material becomes hard^24,25^. All these factors make Al alloy stronger and more surface indentation resistance.

#### Creep properties

**Figure 5:**
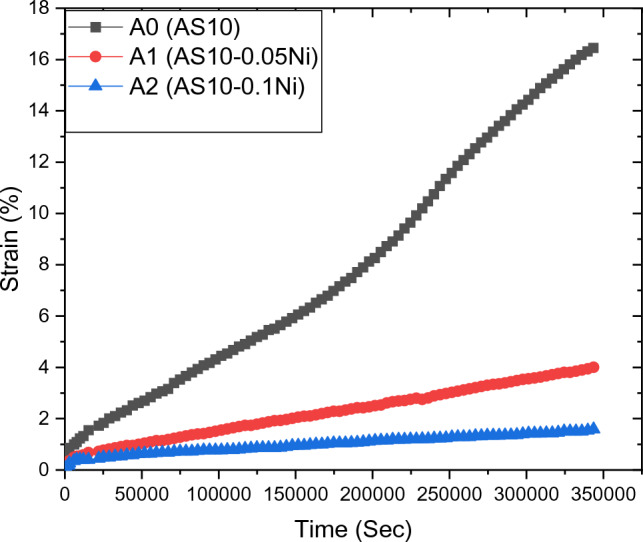
Isothermal creep curve of a constant applied stresses 45 Mpa.

**Figure 6:**
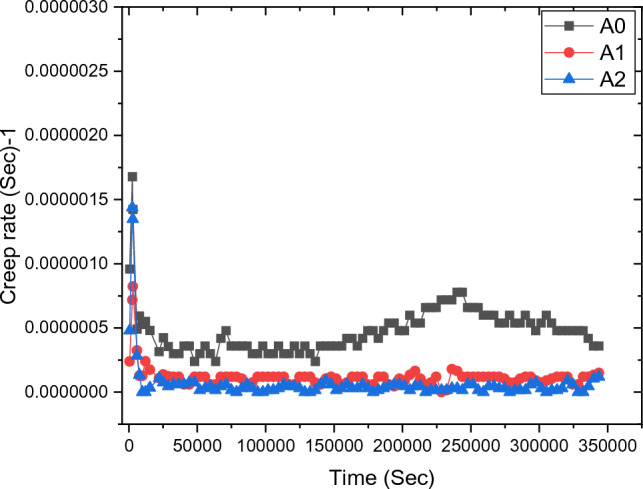
Relation of creep rate versus creep time for AS10-xNi alloys.

**Figure 7:**
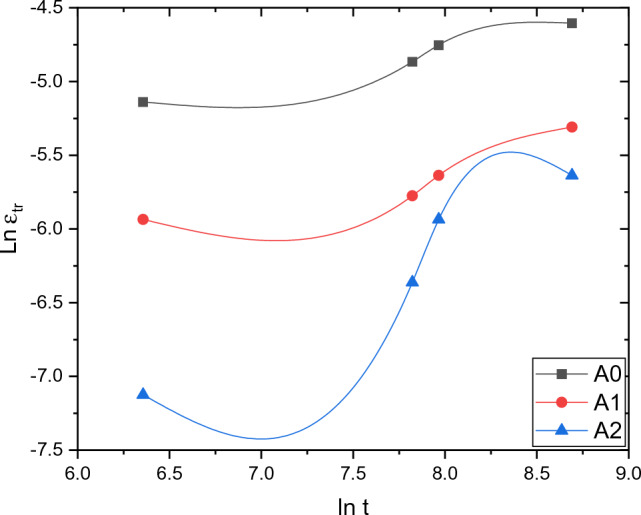
Relation between (Ln Ɛ_tr_) and (ln time) for AS10 at different Ni content and stress 45 Mpa.

**Figure 8:**
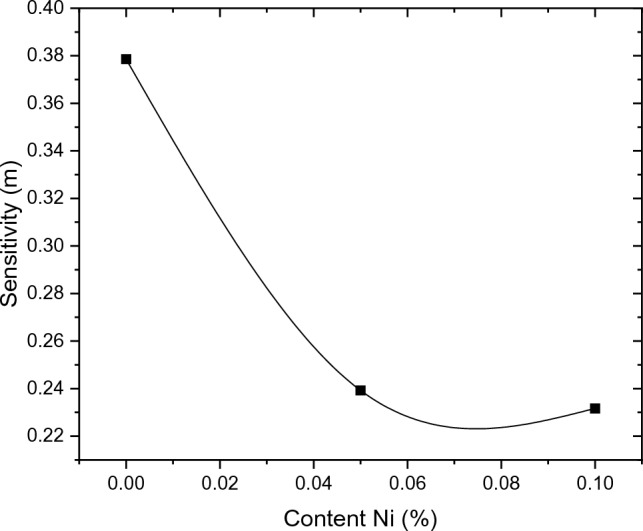
Relation of strain -rate sensitivity with Ni content.

**Figure 9:**
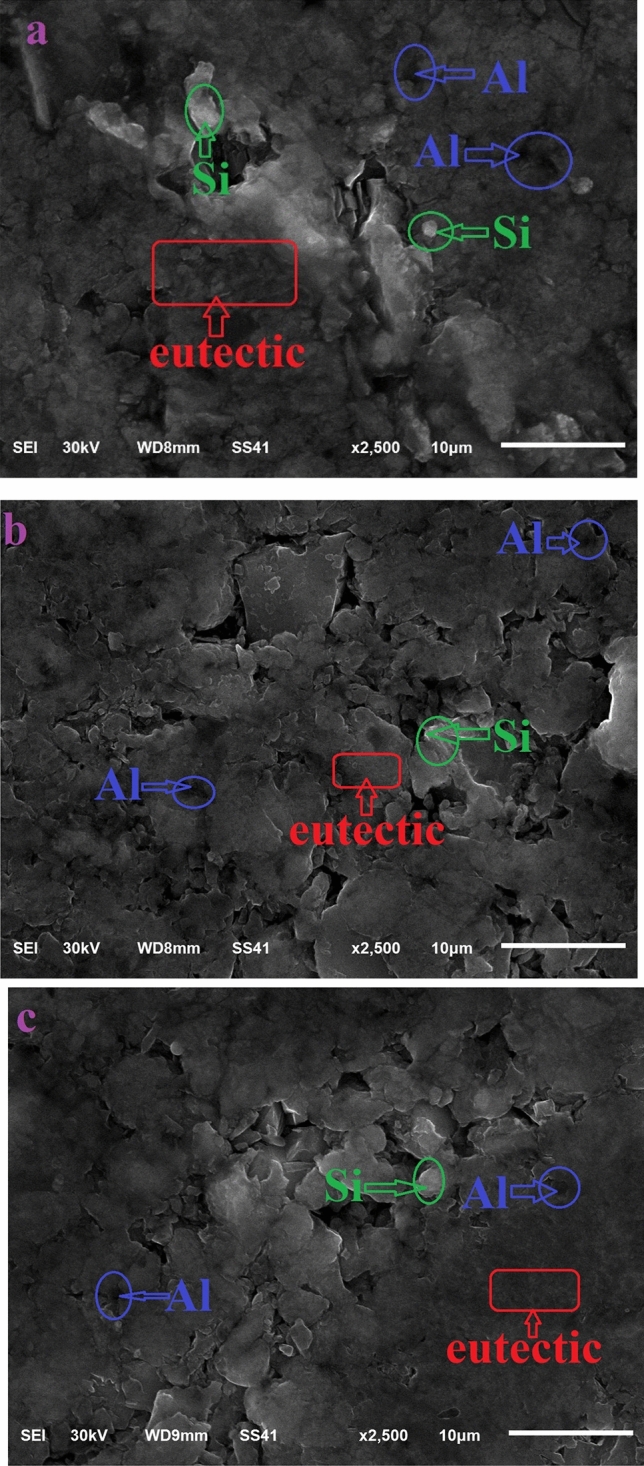
SEM images of the (**a**) hypo-eutectic AS10, (**b**) Ni added (500 ppm) and, (**c**) Ni added (1000 ppm).

**Figure 10:**
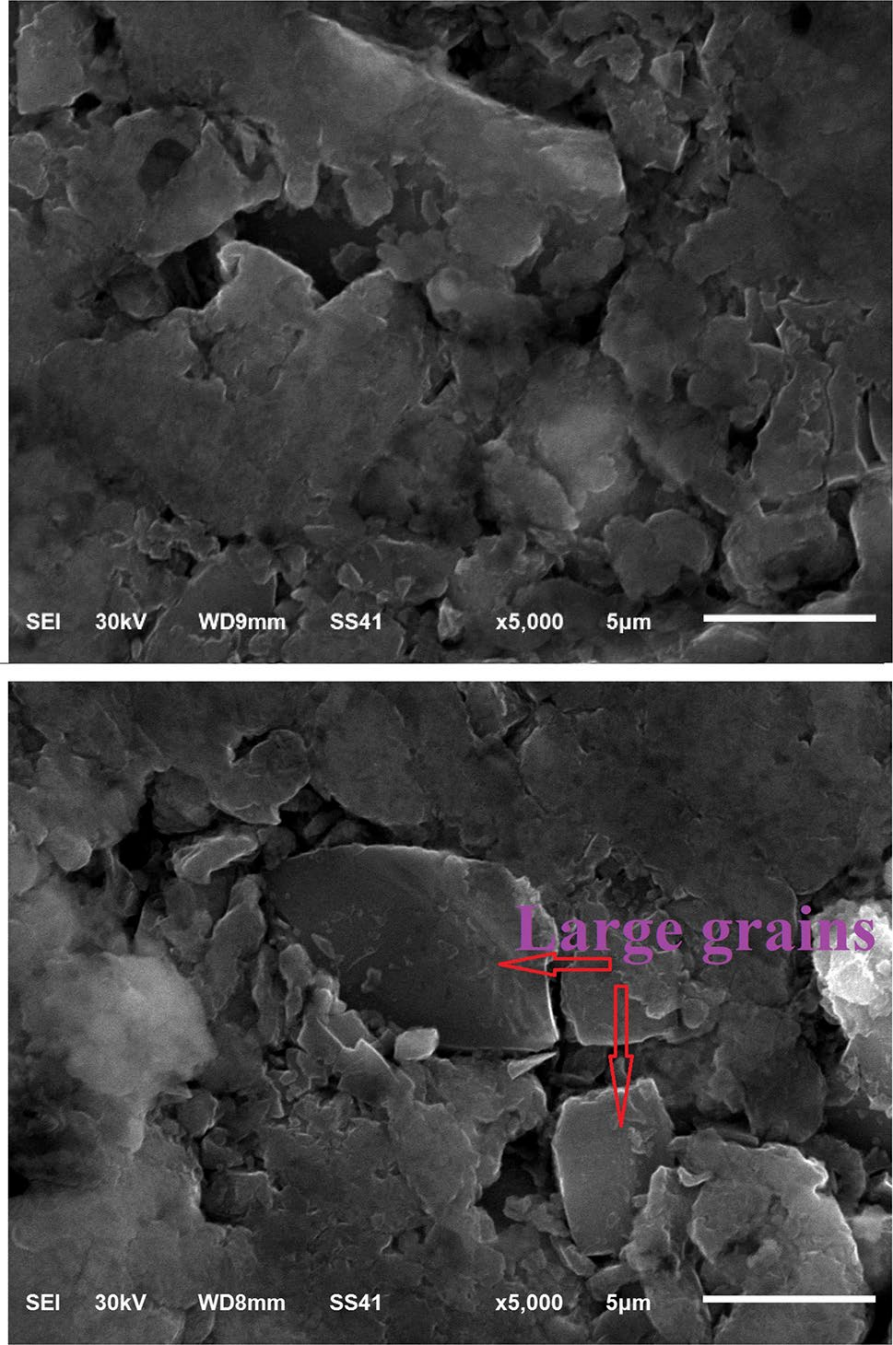
Different grain shapes due to compaction and sintering.

**Figure 11:**
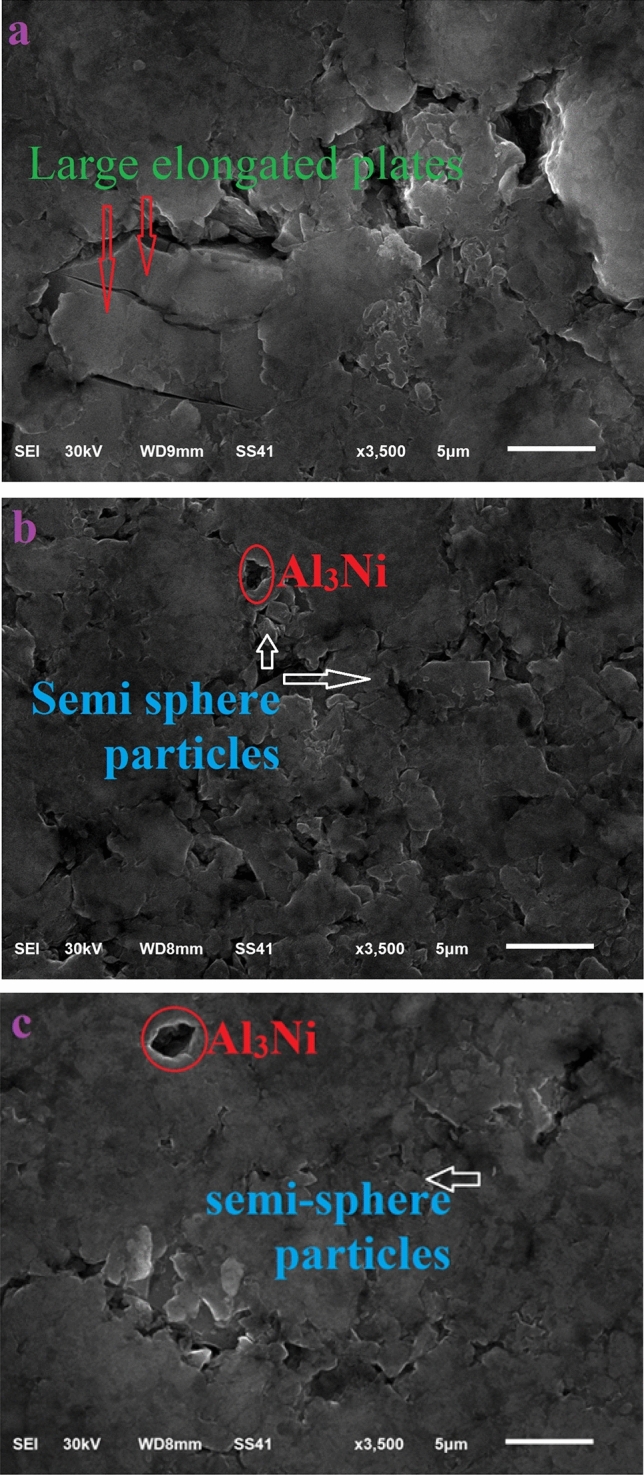
Binary and ternary alloys form.

**Figure 12:**
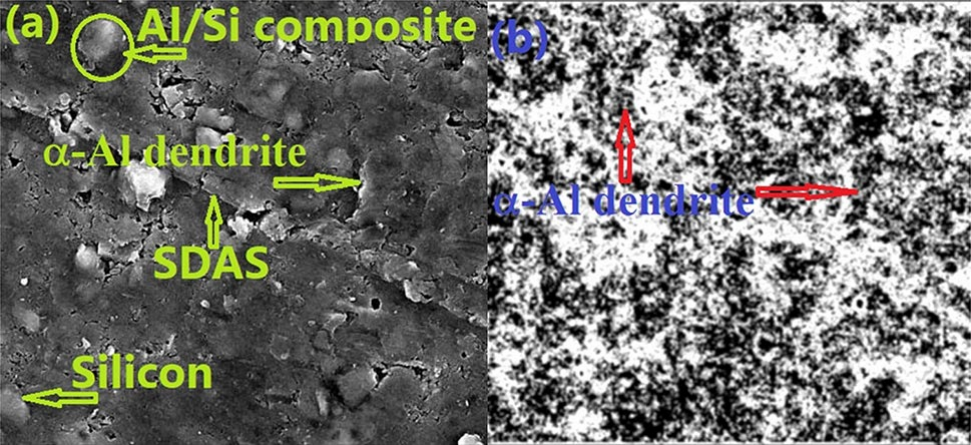
The distinctive dendritic structure of the Aluminium particles.

Isothermal creep curves for the tested alloys were studied by applying a stress of 45 Mpa and a deformation temperature of 298 K. The effects of Ni alloying element additions on the mechanical performance of Al/Si-based alloy are described as creep behavior (Fig. 5). As shown in this Figure, there are three significant regimes: the first regime represents the primary creep. In this regime, the strain is relating with time by the formula, Eq. (3). As testing time increases, the transient strain also increases with a deceleration rate (Figs. 6, 7, 8, 9 and 10). This behavior may be due to two main reasons, (I) residual dislocation motion in the microstructure, (II) static friction stress (σ_0_). The second constant creep rate regime is called the steady-state creep region. In this region, the dislocation motion is uniform and the creep parameters of the materials are determined during this regime as exhibited in Eq. (5). After this region, the nucleation of necking regions is expected^26^. In the necking regions, the stress concentration becomes more and finally leads to failure as observed in the third regime in Fig. 5. In the same Figure, the minimum creep rate decreases with increasing Ni-content as observed through the variation of slope of steady-state regions. The AS10-0.1Ni alloy exhibits the highest creep resistance (low creep rate). This improvement in creep resistance is attributed to the presence of Al_3_Ni, as shown in SEM Fig. 11 b,c which spreads across particles porosities and grain boundaries and does not allow more dislocation movement to occur and then lower creep rate^27^. This distribution of Al_3_Ni helps to resist dislocation motion and finally leads to a lower creep rate. It is found that with increasing nickel content, the average particle size decreases^28^. As the nickel content increases, the diffusion of Ni atoms in the Al lattice increases. This diffusion leads to the growth of aluminum dendrite arms^29^, particularly the secondary dendrite arms (SDA) as shown in Fig. 12.

During solidification, nickel element promotes the formation of smaller and uniform grains. This refinement of the microstructure lead to improve mechanical properties, including higher strength and better resistance to creep deformation^30^.

In order to specify the effect of deformation time on the creep-strain acceleration, the data of the relation-ship between creep strain rate and time have been extracted from Fig. 5 and replotted in Fig. 6.

In order to identify the mechanism of creep deformation, the activation energy of deformation has been calculated and compared with the reference map^31^. The activation energy was calculated from Eq. (9) and tabulated in the Table 3. From this table and Fig. 6, it can be concluded that when adding Ni element to Al/Si alloy, the secondary creep rate decreases while the creep lifetime in this stage increases. The activation energy increases with increasing nickel content and this indicates that the material’s resistance to deformation is improved. This means that as the Ni-content increased as the lifetime of the material increased. This refers to the enhancement in creep resistance and life time^32^.

**Table 3:** Comparison of the steady – state creep characteristics of the tested alloys.

Materials	m	ε̇_st_	Q(KJ mol^−1^)
A_0_	0.37861	4.3E−5	25
A_1_	0.23927	1.23E−7	39
A_2_	0.23168	7.31E−8	40

Figure 7 shows the transient creep behavior as a plot of lnƐ_tr_ against lnt. From the curves, the slope represents time exponent (n) while the intercept represents (β) parameter. All these parameters are shown in the Table 4 as a function of Ni-content. The exponent (n) was found to have values ranging from 0.222 to 0.653. These values obtained are in well consistent with the values reported by Salem^18^.

**Table 4:** Comparison between transient creep parameters for the tested alloys.

Materials	n	β	ɣ
A_0_	0.22266	0.00136	0.656461785
A_1_	0.1545	9.51289E−4	0.437330623
A_2_	0.65394	1.12447E−5	0.693559402

The steady-state strain rate ε̇_st_ of the alloys has been calculated from the gradient of the regular parts of the acquired creep curves in Fig. 5. According to the Eq. (8), the mechanical response of materials is determined via the sensitivity for deformation loads. This sensitivity parameter has been calculated from the slope of the steady-state regions of creep curves in Fig. 5 and tabulated as a function of Ni-content as shown in Fig. 8.

### 4–2 Scanning electron microscope

In order to investigate the microstructure and the effects of Ni addition on the characteristics of AS10 alloy, morphology and topography have been conducted on unmodified and modified specimens by secondary electrons (SE) and backscattered electrons (BSE) images produced by scanning electron microscopy as shown in Fig. 9. It’s well known, that the microstructure of Al/Si/Ni contains many different phases and has a finer structure than that containing only one or two phase^33^. This phenomenon has been expected and detected in this work when modifying Al/Si binary composition by adding traces of Ni-atoms into AS10 binary. These changes in microstructure size are very clear when comparing between Fig. 9a–c. In Fig. 9, it can be seen that there are three regions contained in the overall structure. The first region is the elementary Al powder large particles with a black-gray color. The second region is the master alloy which consists of primary silicon surrounded by the third region of the eutectic Al-Si structure with white grey.

Because the force increases gradually during compaction, the nanocomposite structure begins to produce a sandwich structure Fig. 9. This occurs due to the collision of particles during compaction. During sintering, this structure easiest the welding of the particles together and turns to produce rough surfaces with coarse grains (large size) as can see in Fig. 10. The successive welding of the particles continues as a result of the sintering treatment. This leads to making a dense structure and so more hardness. It can be seen that the primary silicon is independent in the Al-Si alloy. In many regions but not all, it can be seen cracked silicon particles. This observation may due to the indentation region that has been occurred during testing. In the unmodified and modified alloys, there are many porosities uniformly distributed in the overall structure.

If one compares binary and ternary alloys from Fig. 11a–c, respectively, he will detect that in binary composition the particles are in the form of elongated plates with large sizes whereas they are small elongated plates and semi-sphere particles in the ternary composition. This reduction in the size of the modified alloy is attributed to the parts of million of Ni atoms that are added to the alloys. From the Al/Ni binary equilibrium phase-diagram, Al and Ni atoms engaged in a chemical reaction and formed Al_3_Ni IM’C-phase. It can be observed that Al_3_Ni is included in the structure as small black particles are distributed randomly in the microstructure as shown in Fig. 11c. The key factor behind HV’s improvement is this new phase. Like ceramic materials, any chemical compound works, as a dislocation slip resistance, so the dislocation slip is destroyed and the material becomes harder^34,35^. These factors resist the indentation of the Al surface. Figure 12b showcases an image that has been processed using ImageJ software, clearly highlighting the distinct dendritic structure of the Aluminum particles^36^.

## Conclusion

The present research investigates the mechanical properties and microstructure of Al-10%Si added to it 0.05% and 0.1% Ni. The ternary alloy has a higher hardness than the binary one due to the formation of the chemical compound Al_3_Ni. The variation in slopes of steady-state areas shows that the minimum creep rate reduces with increasing Ni concentration. The highest creep resistance is found in the AS10-0.1Ni alloy (low creep rate). Al_3_Ni, which distributes across grain boundaries, prevents further dislocation movement, which in turn lowers the creep rate, and is responsible for this improvement in creep resistance. The activation energy rises as nickel concentration rises, which suggests that the material has better deformation resistance. This indicates that the material’s lifetime rose as the Ni-content rose. If one compares the binary and ternary alloys, from SEM photos it will see that the silicon powder particles in the ternary composition are smaller, and semi-spheres. The addition of Ni atoms to the alloys may be to blame for this reduction in the size of the modified alloy’s structure.

## Data Availability

The authors confirm that the data supporting the findings of this study are available within the article.
